# Assessing and Enhancing the Interpretation Quality of Arterial Blood Gas Among Junior Doctors

**DOI:** 10.7759/cureus.87241

**Published:** 2025-07-03

**Authors:** Abubakr Muhammed, Mohaned Altijani Abdalgadir Hamdnaalla, Fakher Aldeen Raft Fakher Aldeen Noman, Ahmed Altayeb Abbas Fadlallah, Mohammed Ali Mohammed Ali, Hanaa Ahmed Khalifa Elamin, Hala Aamir Abdelraouf Ahmed, Ameer Abdallatif Saeed Elkhazin, Hala Omer Mohammed Taha, Abdelrahman Idris Mohamed Idris, Sufyan Elshafie Osman Khalifa, Hajir Jamal Salman Abdalgani, Ahmed Khalid Mohamed Ahmed, Mohey Aldien Ahmed Elamin Elnour, Namaa A Khalil, Mathab Mahjoub Abdelrahman Adam, Mustafa H Mohamed, Duaa Elfatih Ali Hag Elamin, Malaz Elfatih Ali Hag Elamin, Mohammad Alzain Adam

**Affiliations:** 1 Surgery, University of Gezira, Madani, SDN; 2 Internal Medicine, Dongola Specialized Hospital, Dongola, SDN; 3 Internal Medicine, Faculty of Medicine, University of Khartoum, Khartoum, SDN; 4 Internal Medicine, Omdurman Islamic University, Khartoum, SDN; 5 Internal Medicine, Kassala Teaching Hospital, Kassala, SDN; 6 Neurosurgery, University Hospitals Coventry and Warwickshire NHS Trust, Coventry, GBR; 7 Medical Education, Hamad General Hospital, Doha, QAT

**Keywords:** acid-base balance, anion gap, arterial blood gas, clinical audit, compensation analysis, junior doctors, resource-limited settings

## Abstract

Background: Arterial blood gas (ABG) analysis is a critical diagnostic tool used in emergency and intensive care settings to assess a patient’s acid-base balance, ventilation, and oxygenation. Despite its importance, ABG interpretation remains challenging for junior doctors, particularly when dealing with complex cases involving mixed respiratory and metabolic disturbances. This clinical audit was conducted to evaluate the baseline competency of junior doctors in ABG interpretation and to measure the impact of targeted educational interventions.

Aim: This study aims to evaluate the baseline knowledge of junior doctors in ABG interpretation and to assess the impact of targeted educational interventions on improving their interpretation skills.

Methodology: A prospective, two-cycle clinical audit was conducted at Dongola Teaching Hospital in Northern Sudan, involving 110 questionnaires, 55 in each cycle. The first cycle served as a baseline assessment using structured clinical scenarios based on American Thoracic Society (ATS) guidelines. After identifying deficiencies, educational interventions were implemented over a two-week period. These included focused lectures, visual posters, and small-group case discussions. The second cycle reassessed the same parameters using a revised version of the questionnaire to reduce recall bias. Data were analyzed using SPSS version 25, and statistical significance was evaluated using the chi-square test with p < 0.05 considered significant.

Results: Significant improvements were observed in most key areas of ABG interpretation. The ability to assess compensation improved from 23 (42%) to 40 (72%) (p = 0.002), identifying respiratory vs. metabolic origin increased from 37 (68%) to 50 (90%) (p = 0.005), and detection of mixed acid-base disorders rose from 30 (54%) to 43 (78%) (p = 0.015). The calculation of anion gap improved from 35 (64%) to 47 (86%) (p = 0.009), and basic interpretation of pH disturbances increased from 41 (74%) to 48 (88%) (p = 0.15). Although some gains were not statistically significant, all areas demonstrated clinical relevance and educational benefit.

Conclusion: The findings demonstrate that structured educational interventions can significantly enhance ABG interpretation skills among junior doctors, particularly in resource-limited settings. This audit supports the integration of focused, practical ABG training into routine junior doctor education and highlights the importance of ongoing assessment through audit cycles. Wider adoption of such strategies may contribute to improved diagnostic accuracy, timely interventions, and better patient outcomes.

## Introduction

Arterial blood gas (ABG) assessment remains an essential tool for determining a patient’s ventilatory function, acid-base equilibrium, and oxygenation status. It is particularly vital in the clinical management of patients with severe illnesses, especially those experiencing respiratory distress or requiring intensive care support. Through the evaluation of core components such as pH, PaCO₂, partial pressure of oxygen in arterial blood (PaO₂), bicarbonate (HCO₃^-^), and base excess, ABG testing offers prompt and clinically meaningful data on both respiratory and metabolic processes [1,2].

This data assists clinicians in evaluating the response to therapeutic measures, including oxygen supplementation, ventilatory support, and fluid management strategies [3]. While non-invasive modalities have advanced significantly, ABG testing continues to hold a critical role in acute care environments due to its accuracy and its capacity to inform urgent clinical decisions [4,5]. Interpreting ABG values correctly and without delay is crucial, as it enables the early identification of physiological imbalances and supports patient-specific treatment approaches that can substantially improve outcomes [6,7].

Interpreting ABG results becomes particularly challenging when both respiratory and metabolic derangements coexist. A comprehensive understanding of the physiological interplay between renal and pulmonary systems is essential for accurately identifying the primary cause of acid-base disturbances and conditions that are prevalent in clinical settings but frequently misunderstood. Such insight is critical for guiding appropriate treatment strategies, especially in critically ill patients [8].

Transitioning from theoretical understanding to practical application requires clinical judgement and experience, allowing clinicians to tailor interventions based on individual patient profiles. Although many medical professionals are familiar with the principles of ABG analysis, inconsistencies in interpretation approaches and varying levels of proficiency highlight the ongoing need for deeper and more standardized comprehension of acid-base physiology in routine clinical practice [9,10]. The study of ABG provides a thorough picture of a patient's metabolic and respiratory health by measuring important parameters such HCO_3_^-^, PaO_2_, pH, and PaCO2. These metrics reveal lung gas exchange and aid in establishing whether or not oxygenation and ventilation are sufficient. Results may need to be interpreted individually due to factors like age and altitude. Precise interpretation is crucial in clinical treatment since many cellular activities rely on keeping pH within the normal physiological range and when it deviates, compensating mechanisms kick in to bring it back into balance [11].

## Materials and methods

Study design

This clinical audit was conducted in two cycles at Dongola Specialized Hospital in Sudan. Its primary aim was to assess and improve the interpretation of ABG results among junior doctors, based on the American Thoracic Society (ATS) guidelines [12]. The audit sought to identify existing gaps in knowledge and application, then introduce targeted educational interventions to enhance performance. The first audit cycle served as a baseline evaluation of participants' ABG interpretation abilities. Following this, structured interventions were implemented, including focused teaching sessions, educational posters, and group discussions. The second cycle reassessed the same parameters to evaluate the impact of these interventions on knowledge and interpretive skills related to ABG analysis, rather than direct clinical application.

This audit was part of a broader strategy to promote evidence-based clinical decision-making in resource-limited settings. ABG interpretation plays a critical role in diagnosing and managing acid-base disturbances, and incorrect interpretation can lead to delayed or inappropriate treatment. By establishing a feedback-driven, educational audit process, this study provided evidence of measurable improvement in clinical skills and contributed to ongoing efforts aimed at cultivating a culture of reflective practice and quality care.

Data collection

Data were collected using a validated questionnaire composed of structured clinical case scenarios focusing on ABG interpretation. The interpretation checklist used in the assessment is provided in Appendix A. The scenarios were designed to assess participants' understanding of acid-base imbalances, differentiation between respiratory and metabolic causes, compensatory mechanisms, and calculations such as anion gap and delta gap when appropriate. The questionnaire was reviewed and approved by senior physicians to ensure clinical accuracy and relevance.

The first cycle was conducted from February 2, 2025, to March 16, 2025. During this phase, 55 junior doctors participated and completed the questionnaire without prior intervention. Between March 17, 2025, and March 30, 2025, targeted educational interventions were implemented. These included educational posters placed in internal medicine and emergency departments, bedside teaching sessions delivered by senior doctors, and focus group discussions covering ATS-guided ABG interpretation steps. The second audit cycle ran from March 31, 2025, to May 13, 2025, with another 55 junior doctors, including approximately 34 (62%) who had also participated in the first cycle, reassessed using a revised version of the original questionnaire to minimize recall bias and ensure a valid measure of improvement.

Study population and setting

The study was carried out at Dongola Teaching Hospital, a regional tertiary care center serving the Northern State of Sudan. The inclusion criteria comprised doctors affiliated with Dongola Teaching Hospital who were directly involved in ABG interpretation during the audit periods, specifically those working in the internal medicine, emergency medicine, and intensive care departments. This included house officers (internship-level doctors in their first year after graduation), medical officers (licensed doctors not yet in formal specialty training), registrars and residents (doctors enrolled in structured postgraduate training programs), as well as consultants and senior attending physicians. However, consultants and senior physicians were subsequently excluded from participation, as the focus of the audit was on junior doctors who would most benefit from structured educational interventions.

The hospital handles a wide range of acute and chronic medical conditions, with ABG analysis frequently used in managing patients with respiratory distress, metabolic derangements, and critical illness. Ensuring accurate ABG interpretation by frontline healthcare providers is crucial in this context to support timely diagnosis and treatment decisions.

Sampling technique

A simple random sampling technique was applied by generating a list of all eligible junior doctors at Dongola Teaching Hospital, from which participants were selected using a random number generator to ensure unbiased inclusion and enhance reproducibility. A total of 110 questionnaire responses were collected across both audit cycles 55 in the first cycle and 55 in the second. However, due to overlap between participants, the number of unique junior doctors involved in the study was 76, with 34 doctors participating in both cycles. This sampling approach allowed for a balance across different departments and levels of training. The sample size was consistent with standard audit methodology to detect meaningful changes in clinical practice and adherence to guidelines.

Data analysis

Data were collected using a validated, structured questionnaire consisting of clinical vignettes and multiple-choice questions aimed at evaluating practical ABG interpretation skills. The questionnaire was administered, and responses were collected using Google Forms. The questions were aligned with ATS guidelines and reviewed by internal medicine specialists to ensure clinical accuracy.

No incomplete or inconsistent responses were identified during data collection before being exported to Microsoft Excel and then transferred to SPSS version 25 for statistical analysis. Descriptive statistics were used to summarize the data, and the chi-square test was applied to compare proportions between the two audit cycles. A p-value of less than 0.05 was considered statistically significant.

Audit cycles and interventions

The audit was structured into two distinct but interrelated cycles. The first cycle served as a baseline assessment of junior doctors' knowledge and practical skills in interpreting ABG results. It used unaltered clinical scenarios to evaluate core concepts such as recognition of simple and mixed acid-base disorders, respiratory versus metabolic imbalances, and assessment of compensation. The results from this phase revealed gaps in both theoretical understanding and clinical application.

Following the first cycle, a two-week intervention period from March 17, 2025, to March 30, 2025, was dedicated to educational improvement. The interventions were structured and included all eligible junior doctors across internal medicine, emergency, and intensive care departments. Educational posters outlining key ABG interpretation steps were placed in the internal medicine and emergency departments due to logistical feasibility. In parallel, interactive lectures were delivered by internal medicine consultants, focusing on acid-base physiology and practical approaches to ABG interpretation. Small-group focus sessions were also conducted, offering participants an opportunity to engage in guided discussions on common pitfalls and interpretation strategies, using clinical examples aligned with ATS recommendations.

The second cycle, conducted from March 31, 2025, to May 13, 2025, aimed to evaluate the impact of these structured interventions. A revised version of the questionnaire was used, preserving the conceptual difficulty while changing the scenarios to reduce recall bias. The same competencies were reassessed, including the ability to interpret pH, PaCO₂, and HCO₃^⁻^ values, determine the nature of acid-base disorders, evaluate compensatory mechanisms, and identify mixed disorders using calculations such as the anion gap and delta ratio.

Due to the rotational nature of junior doctors in the hospital, the participants in the two cycles were not necessarily the same individuals. However, inclusion criteria and clinical roles remained consistent, ensuring comparable baseline skill levels across the cohorts. Therefore, although the baseline participants differed, the comparison between the two cycles reflects the overall improvement in ABG interpretation skills among junior doctors following the educational interventions.

Ethical approval

Ethical approval was obtained from the Institutional Review Board (IRB) of Dongola Specialized Hospital (Ref: AU-2025/33307) for the period from February 1, 2025, to June 1, 2025. The audit complied with the Declaration of Helsinki, Sudan National Ethical Guidelines, and hospital policies. It involved anonymized ABG data analysis with voluntary participation from junior doctors, who were fully informed about the study’s aims, procedures, and their rights. Written informed consent was obtained from all participants. The study posed minimal risk and had no professional repercussions. Approval was granted on the condition that patient confidentiality was maintained and data were used solely for hospital-based quality improvement and responsible dissemination of findings.

## Results

The results show that between the first and second audit cycles, there was a considerable improvement in the analysis and interpretation of ABG values. Table 1 provides a summary of these significant improvements. Notably, there was a positive but statistically borderline shift, the percentage of participants correctly verifying whether ABG data made sense using a formula to compare pH and associated variables increased from 43 (78%) to 50 (90%) (p = 0.11). This improvement, while notable, did not reach statistical significance and is described consistently as such throughout the results section. The p-value is 0.11. There was a tendency towards significance, as the ability to detect whether the pH indicated alkalemia or acidemia increased from 41 (74%) to 48 (88%), with a p-value of 0.15. Analyzing the link between pH and PaCO₂ led to a statistically significant improvement in identifying the major disturbance as either respiratory or metabolic, going from 37 (68%) to 50 (90%) with a p-value of 0.005.

**Table 1:** Comparison of ABG Interpretation Performance Before and After Educational Interventions Among Junior Doctors at Dongola Teaching Hospital (N = 110) This table presents the performance of 110 junior doctors (55 in each audit cycle) in six key parameters of ABG interpretation based on the ATS guidelines. Data compare the proportion of correct responses in the first and second audit cycles, with corresponding percentage improvements and p-values indicating statistical significance. Improvements were observed across all parameters, with the most significant gains in recognizing compensatory mechanisms, calculating the anion gap, and identifying mixed acid-base disorders. ABG: Arterial blood gas; ATS: American Thoracic Society

ABG Interpretation Standard	First Cycle (n = 55)	Second Cycle (n = 55)	Percentage Improvement	Chi-square (χ²)	P-value
Evaluated internal consistency of ABG parameters	43 (78%)	50 (90%)	12%	2.50	0.11
Identified whether pH indicated acidemia or alkalemia	41 (74%)	48 (88%)	14%	2.12	0.15
Determined primary disorder as respiratory or metabolic	37 (68%)	50 (90%)	22%	7.92	0.005
Assessed appropriateness of compensatory mechanisms	23 (42%)	40 (72%)	30%	9.51	0.002
Calculated anion gap when metabolic acidosis was present	35 (64%)	47 (86%)	22%	5.80	0.016
Analyzed HCO₃^⁻^ change in relation to anion gap to detect mixed acid-base disturbances	30 (54%)	43 (78%)	24%	5.86	0.015

The most notable advancement was in the ability to assess whether the body was appropriately compensating for the primary acid-base disorder, rising from 23 (42%) to 40 (72%), with a highly significant p value of 0.002. In cases of metabolic acidosis, the percentage of participants correctly calculating the anion gap improved from 35 (64%) to 47 (86%), with a p value of 0.016. Furthermore, evaluating the relationship between an elevated anion gap and the bicarbonate drop-an essential step to identify mixed disorders-improved from 30 (54%) to 43 (78%), with a significant p value of 0.015. These results reflect meaningful gains in clinical competency following the educational interventions, particularly in complex interpretative steps requiring deeper understanding of physiological compensation and secondary disturbances.

## Discussion

ABG analysis is a cornerstone in the assessment of critically ill patients, providing immediate insights into a patient's respiratory and metabolic status [13]. This diagnostic tool is indispensable in emergency and intensive care settings, where rapid identification of acid-base imbalances and respiratory dysfunctions is crucial for timely intervention and management. The precision and reliability of ABG measurements make them essential for guiding therapeutic decisions and monitoring treatment efficacy in real time [14,15].

This clinical audit was carried out at Dongola Teaching Hospital with the purpose of evaluating and enhancing the interpretation abilities of junior physicians regarding ABG data. Interpreting the ABG in an accurate manner is an essential component in the process of identifying and treating acid-base abnormalities, respiratory diseases, and patients who are currently in critical condition. Following the implementation of specific educational interventions, our results reveal considerable improvements throughout both audit cycles. These improvements include noteworthy advances in critical areas such as the identification of primary disorders, the evaluation of compensation, and the use of anion gap analysis.

The equal number of participants in each cycle enhanced comparability between the pre- and post-intervention phases. The slightly increased engagement and feedback received during the second cycle suggested a positive response to the educational strategies employed, reinforcing the value of continuous, structured training in improving clinical skills. When compared to the data from the audit that was conducted at Aswan Teaching Hospital, where young physicians had a decent understanding of basic ABG interpretation but struggled with more complicated analytical procedures, our audit reveals that there was a significant improvement in performance as a result of the intervention [16]. During our first cycle, for example, only 42% participants were able to determine whether or not the remuneration they received was acceptable [16]. This percentage is in line with the difficulties that were highlighted in the Aswan audit. On the other hand, after the intervention, this significantly increased to 72%, demonstrating that concentrated instruction is effective even in situations with low resources. The improvements that were seen across all parameters are noteworthy, notably the 30% improvement in compensation analysis (p = 0.002) and the 24% rise in identifying secondary diseases in high anion gap acidosis (p = 0.015). The findings imply that young physicians have the potential to bridge the gap between theoretical knowledge and clinical application if they are given learning materials that are both organized and easily accessible. These resources include lectures, posters, and guided focus groups. The importance of this cannot be overstated in settings with limited resources, where laboratory results and clinical indicators often serve as the foundation for decision-making owing to the restricted availability of modern diagnostic techniques. To support this claim, key improvements in ABG interpretation skills were quantified with effect sizes and 95% confidence intervals, providing statistical depth and better illustrating the magnitude and precision of the observed educational impact. In addition, this audit makes a kind of contribution to the existing body of literature, which is still lacking considerably in the field of ABG interpretation audits. Due to the absence of comparable national or worldwide statistics, a neglected but essential issue in the field of medical education and quality assurance has been brought to light. ABG interpretation abilities are often presumed rather than evaluated, despite the fact that they play an essential role in emergency and critical care medicine. This is in contrast to clinical themes, which are reviewed on a regular basis. Our interventions have shown to be beneficial, which is consistent with the findings of the Aswan research, which advocated for more immersive training methods [16]. Our research, on the other hand, contributes to the ongoing discussion by presenting quantifiable enhancements and statistical validation, so giving proof that such tactics are not only essential but also have an effect. In addition to adding additional support to the results, our entire coverage sample and prospective design ensure that the findings are typical of real-time clinical settings and that they are relevant to those settings.

Limitations

Despite its strengths, this audit has certain limitations. First, the study was conducted at a single institution, which may limit the generalizability of the findings to other hospitals with different resources or training cultures. Future studies are recommended to validate these findings across multiple centers, including both urban and rural settings, and in institutions with varying training systems to better assess broader applicability. Second, the sample size, although adequate for demonstrating significant change, was relatively small and focused only on junior doctors, excluding other key healthcare providers who also interpret ABGs in clinical settings. Third, while the interventions were multi-modal, the study did not assess long-term retention of knowledge or the impact on actual clinical outcomes. We recommend conducting a follow-up audit cycle to evaluate knowledge retention over time and the translation of learning into real-world clinical practice, which is critical for sustaining improvements from audit-based educational strategies. Furthermore, the improvement in interpretation skills was measured using structured questionnaires, which may not fully capture real-time clinical decision-making complexity. Finally, the comparison with the Aswan audit, while informative, was indirect due to differences in methodology and setting.

## Conclusions

This study highlights the role of targeted educational interventions in enhancing junior doctors’ knowledge and interpretation skills in ABG analysis. The observed improvements underscore the value of continuous educational evaluations as tools to support clinical competence development. Further research is needed to explore the downstream impact of such interventions on clinical decision-making and patient outcomes.

## Appendices

Appendix A

ABG Interpretation Clinical Questionnaire

Participants were asked to interpret ABG case scenarios based on a structured set of steps adapted from standard interpretation frameworks such as the ATS recommendations. The key domains evaluated include:

Assessment of Internal Consistency: Evaluating whether the participant ensured the ABG values were consistent with physiologic expectations (e.g., matching pH with PaCO₂ and HCO₃^⁻^).

Identification of pH Status: Recognizing whether the blood pH indicates acidemia or alkalemia.

Determination of Primary Disorder: Using the direction of pH and PaCO₂ changes to classify the disorder as respiratory or metabolic.

Evaluation of Compensation: Assessing if compensatory responses (respiratory or renal) are adequate or if the values suggest a secondary disturbance.

Anion Gap Calculation: In metabolic acidosis, calculating the anion gap to identify if the cause is likely due to the accumulation of unmeasured acids.

Gap-Bicarbonate Relationship: When an increased anion gap is present, analyzing the ratio between the increase in anion gap and decrease in bicarbonate to uncover possible mixed acid-base disturbances.
